# Evidence that fodipir (DPDP) binds neurotoxic Pt^2+^ with a high affinity: An electron paramagnetic resonance study

**DOI:** 10.1038/s41598-019-52248-9

**Published:** 2019-11-01

**Authors:** Jan Eric Stehr, Ingemar Lundström, Jan Olof G. Karlsson

**Affiliations:** 1 Department of Physics, Chemistry and Biology, Linköping University, Linköping, Sweden; 2 Division of Drug Research/Pharmacology, Linköping University, Linköping, Sweden

**Keywords:** Biophysics, Oncology

## Abstract

Oxaliplatin typically causes acute neuropathic problems, which may, in a dose-dependent manner, develop into a chronic form of chemotherapy-induced peripheral neuropathy (CIPN), which is associated with retention of Pt^2+^ in the dorsal root ganglion. A clinical study by Coriat and co-workers suggests that co-treatment with mangafodipir [Manganese(II) DiPyridoxyl DiPhosphate; MnDPDP] cures ongoing CIPN. These authors anticipated that it is the manganese superoxide dismutase mimetic activity of MnDPDP that explains its curative activity. However, this is questionable from a pharmacokinetic perspective. Another, but until recently undisclosed possibility is that Pt^2+^ outcompetes Mn^2+^/Ca^2+^/Zn^2+^ for binding to DPDP or its dephosphorylated metabolite PLED (diPyridoxyL EthylDiamine) and transforms toxic Pt^2+^ into a non-toxic complex, which can be readily excreted from the body. We have used electron paramagnetic resonance guided competition experiments between MnDPDP (^10^logK_ML_ ≈ 15) and K_2_PtCl_4_, and between MnDPDP and ZnCl_2_ (^10^logK_ML_ ≈ 19), respectively, in order to obtain an estimate the ^10^logK_ML_ of PtDPDP. Optical absorption spectroscopy revealed a unique absorption line at 255 nm for PtDPDP. The experimental data suggest that PtDPDP has a higher formation constant than that of ZnDPDP, i.e., higher than 19. The present results suggest that DPDP/PLED has a high enough affinity for Pt^2+^ acting as an efficacious drug in chronic Pt^2+^-associated CIPN.

## Introduction

Oxaliplatin-associated chemotherapy-induced peripheral neuropathy (CIPN) is the most frequent cause of complete discontinuation of an otherwise successful chemotherapy in colorectal cancer (CRC) patients. Chronic CIPN is characterized by bilaterally symmetrical sensory symptoms, e.g., numbness, tingling, and pain appearing in the feet and hands. At present there is no approved therapy for CIPN. The exact mechanism behind oxaliplatin-associated CIPN is poorly understood but it is associated to retention of Pt^2+^ in the dorsal root ganglion (DRG) and subsequent binding to proteins, resulting in increase in cellular oxidative and nitrosative stress^1^. Mangafodipir (Manganese(II) DiPyridoxyl DiPhosphate; MnDPDP), after being metabolized into cell-permeable Manganese(II) Pyridoxyl EthylDiamine (MnPLED), presumably attacks cellular oxidative and nitrosative stress at three levels^1,2^: (i) it lowers the intracellular level of O_2_^•−^ directly through its manganese superoxide dismutase (MnSOD)-mimetic activity; (ii) it binds and disarms endogenous transition metals, particularly Fe^2+^/Fe^3+^ and Cu^+^/Cu^2+^, and thus arrests incorporation of ^•^NO_2_ into tyrosine 34 and a subsequent irreversible inactivation of the mitochondrial MnSOD and production of highly toxic OH^•^ radicals; and (iii) it replaces the irreversible inactivated MnSOD. By these properties, MnDPDP effectively switches off the vicious circle that eventually may cause severe tissue damage^2^.

In its catalytic activity complexed Mn^2+^ of the MnSOD mimetic is first oxidized by O_2_^•−^ to Mn^3+^. Subsequently, the formed Mn^3+^ is reduced to Mn^2+^ by a second O_2_^•−^, making the reaction a true metal-catalyzed dismutation like that catalyzed by the SOD enzymes. Free Mn^2+^ ions in non-complexing buffers, by contrast, are poorly reactive^3^ with O_2_^•−^.

**Figure 1:**
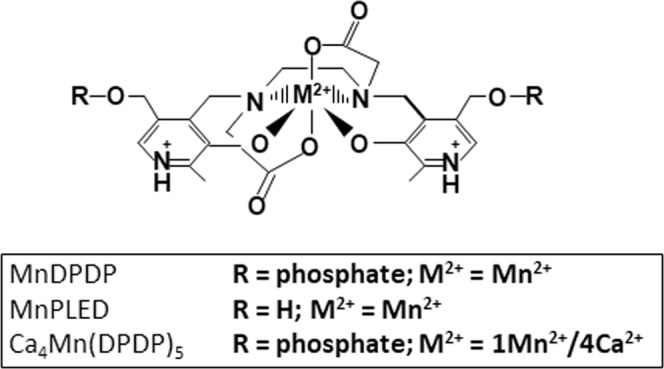
Chemical structure of manganese dipyridoxyl diphosphate (MnDPDP; generic name mangafodipir), manganese pyridoxyl ethyldiamine (MnPLED) and tetracalcium monomanganese penta(dipyridoxyl diphosphate) [Ca_4_Mn(DPDP)_5_; calmangafodipir].

Recent publications by Coriat and coworkers^4^, Glimelius *et al*.^5^, and a prior case report by Yri *et al*.^6^ describe that co-treatment with intravenously administered MnDPDP or Ca_4_Mn(DPDP)_5_ (tetraCalcium monoManganese(II) penta(DiPyridoxyl DiPhosphate) (Fig. 1) may reduce oxaliplatin-associated CIPN in CRC patients. Importantly, the data from the Coriat *et al*. study^4^ suggest that MnDPDP not only prevents but surprisingly also cures chronic CIPN. This particular study showed that patients with pre-existing oxaliplatin-associated CIPN (grade 2 or worse on a 1–3 scale) improved after combined oxaliplatin and MnDPDP treatment. Because the severity of oxaliplatin-associated CIPN correlates with the cumulative dose, continued treatment with oxaliplatin alone is expected to aggravate CIPN. Combined treatment is hence in the best case expected to prevent further aggravation. However, the above cited publications take for granted that it is the MnSOD-mimetic activity of MnDPDP, as described by Asplund *et al*.^7^, Brurok *et al*.^8^ and Bedda *et al*.^9^, that explains its therapeutic activity against chronic oxaliplatin-associated CIPN. *Id est*, in a similar manner as it protects against myocardial injuries caused by ischemia-reperfusion^10^ and doxorubicin-induced cardiotoxicity^11^, chemotherapy-induced myelosuppression^12–14^, and paracetamol (acetaminophen)-induced liver failure, respectively^9^.

The *in vivo* protection of MnDPDP^12,14^ or Ca_4_Mn(DPDP)_5_^14^ against oxaliplatin-induced myelosuppression in mice takes place without interfering negatively with the tumoricidal activity of oxaliplatin. Contrary, these studies^12,14^ showed that both MnDPDP and Ca_4_Mn(DPDP) increased the tumoricidal activity of oxaliplatin. In *vitro* studies^14^ suggest that the increased tumoricidal activity of MnDPDP and Ca_4_Mn(DPDP)_5_ is an inherent property of DPDP/CaDPDP alone and not of the intact Mn^2+^ complex. Glimelius *et al*.^5^ reported that Ca_4_Mn(DPDP)_5_ did not interfere negatively with oxaliplatin-containing chemotherapy in colorectal cancer patients.

An externally administered MnSOD mimetic is expected to result in only a transient increase in MnSOD mimetic activity, mainly governed by the elimination of the MnPLED-derivatives from the body (i.e., MnDPDP, MnDPMP (Manganese(II) DiPyridoxyl MonoPhosphate) and MnPLED). Pharmacokinetic data from human volunteers, as reported by Toft *et al*.^15^, show that very little or no DPDP/DPMP/PLED-bound Mn^2+^ is detected in the blood plasma 2 hours after administration of MnDPDP, whereas ZnPLED is still detected 12 hours after administration. On theoretical grounds a MnPLED-derivative is hence expected to have a preventive efficacy when administered close to the oxaliplatin administration and not a curative efficacy on an already established CIPN. The therapeutic window of MnDPDP and its metabolite MnPLED is likely too short for having any major effect on an already established chronic CIPN, through its MnSOD-mimetic activity. The situation is quite different when it comes to acute and transient increase in reactive oxygen species (ROS) and reactive nitrogen species (RNS) - such as those seen in situations of ischemia-reperfusion injuries, acetaminophen-induced liver failure, chemotherapy-induced myelosuppression, doxorubicin-induced cardiotoxicity, and acute CIPN, where there is little or no doubt that the therapeutic effects are mainly due to the MnSOD-mimetic activity of MnDPDP/Ca_4_Mn(DPDP)_5_^2^.

When it comes to oxidative and nitrosative stress caused by oxaliplatin-associated Pt^2+^, another but until recently undisclosed possibility^3^ is that Pt^2+^ outcompetes Mn^2+^ for binding to DPDP (or its metabolites DPMP and PLED) and transforms toxic Pt^2+^ into a non-toxic complex which, like other metal-DPDP/DPMP/PLED complexes, can be readily excreted through the kidneys. Cu^2+^ binds with high affinity to DPDP and PLED, with a formation constant (^10^logK_ML_) about 22 for both compounds^16,17^. Cu^2+^ binds with four coordinates in a square pyramidal geometry^16,17^, and has an ionic radius of 57 pm^18^. Similarities between Pt^2+^ and Cu^2+^ with respect to binding geometry and ionic radius of 60 pm^18^, suggest that Pt^2+^ should bind to DPDP/PLED with high affinity. The corresponding affinity of Mn^2+^ for DPDP is approximately seven orders of magnitude lower^17^ than that of Cu^2+^.

It is, however, not an easy task to determine the affinity of Pt^2+^ for hexa-dentate chelators, which is reflected by the apparent absence of formation constants of Pt^2+^ for common hexa-dentate chelators, such as EDTA, in the literature. The main reason is most probably a lack of true water-soluble Pt^2+^salts in addition to slow Pt^2+^ exchange kinetics^19^. K_2_PtCl_4_ is a water soluble compound, which dissolves into two K^+^ cations and a square planar metal complex of Pt(II)Cl_4_. This compound is an important starting material for preparation of other coordination complexes of Pt^2+^ aimed for e.g., medical use. Although the chloride ligands of [PtCl_4_]^2−^ are displaced by many other ligands, the ligand-exchange behavior of Pt(II)complexes is quite slow, which gives them a high kinetic stability^20^ and results in ligand-exchange reactions of minutes to days, rather than microseconds to seconds for many other coordination compounds^19^.

**Figure 2:**
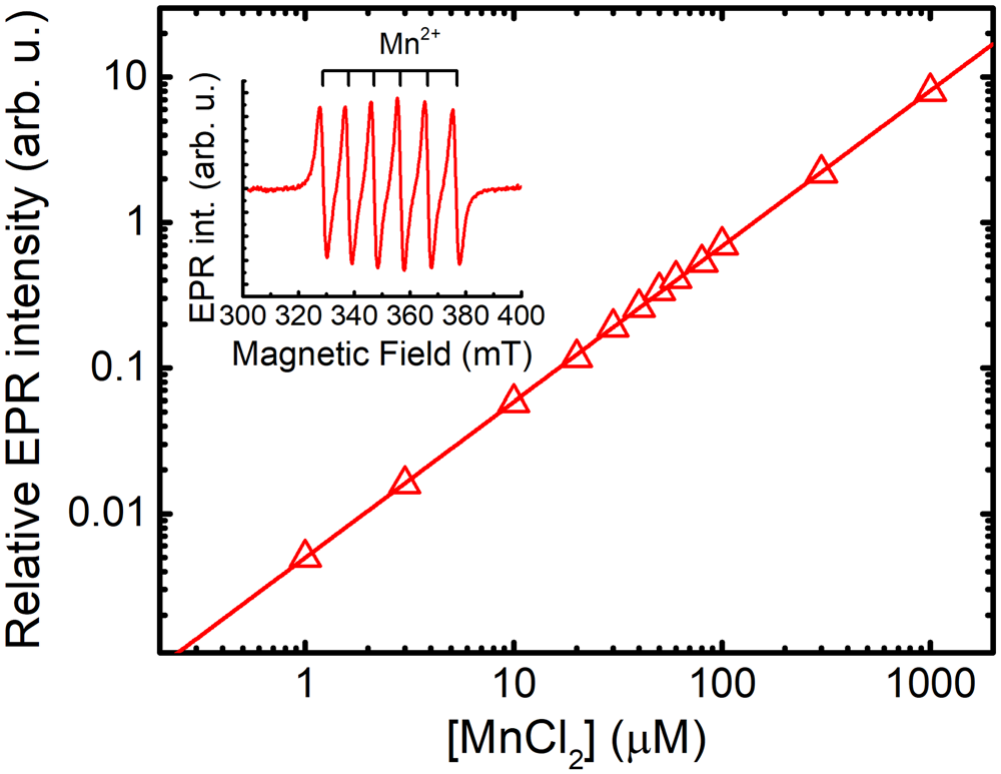
EPR standard curve for free Mn^2+^. The double integral of the EPR signal (insert) in arbitrary units is plotted against the concentration of MnCl_2_.

In the present paper we have used the difference in electron paramagnetic resonance (EPR) spectra of MnDPDP and hexaqua-Mn^2+^ to measure release of Mn^2+^ from DPDP (Fig. 2), as described by Schmidt and co-workers^21^, in exchange for Pt^2+^ and Zn^2+^.

## Results

### EPR-guided competition experiments

**Figure 3:**
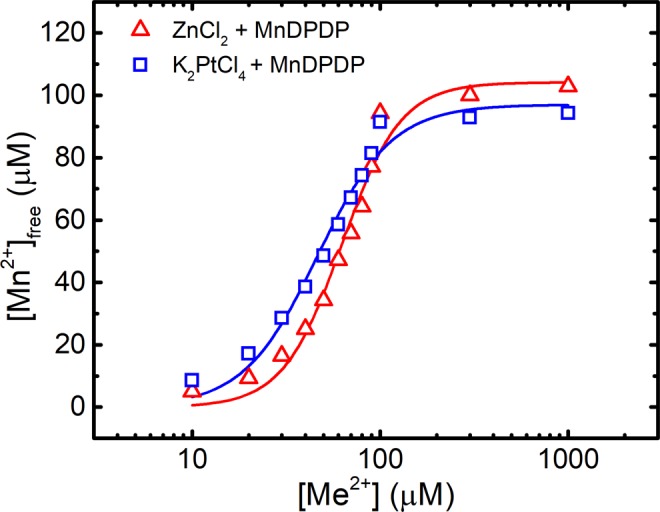
Metal exchange of MnDPDP by Zn^2+^ and Pt^2**+**^. The invisibility of MnDPDP at room temperature due to its high ZFS was used to monitor exchange of Mn^2+^ for Zn^2+^]and Pt^2+^, respectively^21^.

EPR-guided competition experiments between MnDPDP and ZnCl_2_ or K_2_PtCl_4_ are presented in Fig. 3. The resulting competition curve for 100 µM MnDPDP and 10–1000 µM ZnCl_2_ is more or less identical to that presented by Schmidt and co-workers^21^. The corresponding curve for 100 µM MnDPDP and 10–1000 µM K_2_PtCl_4_ lies to the left of the former. The pD_2_ [−^10^log of the concentrations (M) of a drug causing half maximal responses; EC_50_] (together with 95% confidence interval) values for K_2_PtCl_4_ and ZnCl_2_ were 4.280 (4.227–4.332) and 4.173(4.127–4.218), respectively, i.e., there is a statistically significant difference between these two pD_2_ values. This suggests that Pt^2+^ in fact has a higher affinity than Zn^2+^ for DPDP. The present curve for Zn^2+^ and that of Schmidt *et al*. are close to 100% exchange for all concentrations of ZnCl_2_. That means that the stability constant of Pt^2+^ may be substantially higher than that for Zn^2+^. The E_max_ (maximal response) for the K_2_PtCl_4_ and ZnCl_2_ were 95.62 µM (89.54–101.7) and 101.2 µM (95.18–111.2), respectively. There was a clear tendency, although not statistically significant, that the K_2_PtCl_4_ curve did not reach full metal exchange (100 µM). The K_2_PtCl_4_ + MnDPDP samples got a weak yellow-brownish color during incubation. This was not seen in the MnDPDP control sample or in the ZnCl_2_ + MnDPDP samples, which may suggest that Pt^2+^-driven oxidation of Mn^2+^ had occurred to some extent in the K_2_PtCl_4_ + MnDPDP samples, which in turn may explain the somewhat lower E_max_ than the expected.

### Optical absorption spectroscopy

**Figure 4:**
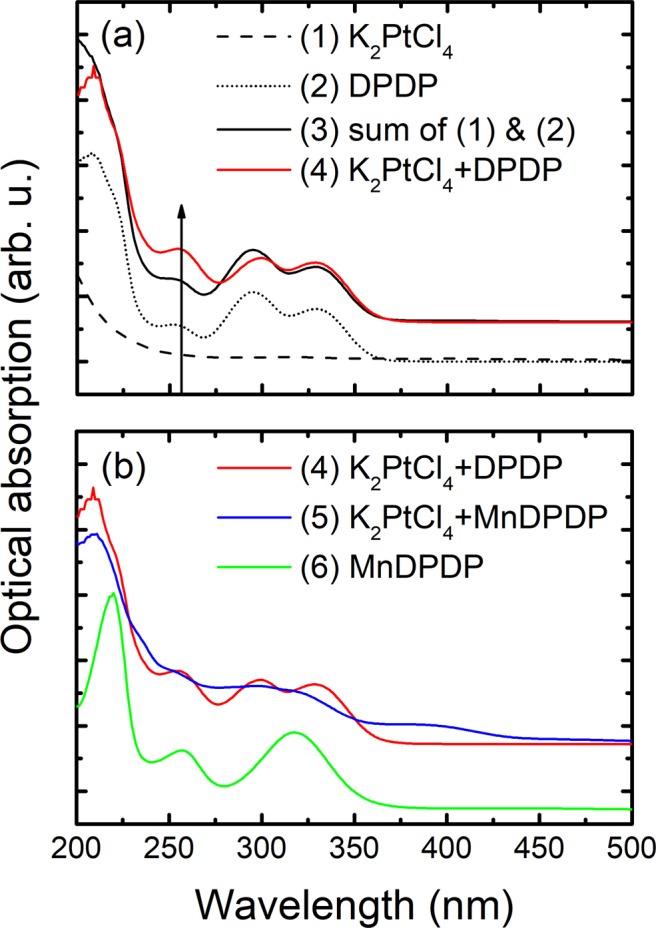
Optical absorption spectra of 100 µM K_2_PtCl_4_ (1), 100 µM DPDP (2), the sum of the spectra of K_2_PtCl_4_ and DPDP (3) and 100 µM K_2_PtCl_4_ + 100 µM DPDP (4) after incubation for 6 h at 60° (spectra 3 and 4 are both offset for clarity) **(a)**. Optical absorption spectra of 100 µM K_2_PtCl_4_ + 100 µM DPDP (4), 100 µM K_2_PtCl_4_ + 100 µM MnDPDP (5) and 100 µM MnDPDP (6) after incubation for 6 h at 60 °C (spectra 5 and 6 are both offset for clarity) **(b)**.

Optical absorption measurements on pure MnDPDP with a 100 µM concentration in aqueous solution show roughly the same UV-absorption maxima at 220, 257 and 319 nm as reported by Tirkkonen *et al*.^22^. 100 µM MnCl_2_ in aqueous solution does not exhibit any peaks in the measured spectral range. The optical absorption measurements in aqueous solutions of 100 µM K_2_PtCl_4_ (1), 100 µM DPDP (2), the sum of spectra of K_2_PtCl_4_ and DPDP (3), 100 µM K_2_PtCl_4_ + 100 µM DPDP (4), 100 µM K_2_PtCl_4_ + 100 µM MnDPDP (5) and 100 µM MnDPDP (6), after incubation for 6 h at 60 °C, are depicted in Fig. 4a,b. A comparison between the sum of the individual optical absorption spectra of 100 µM K_2_PtCl_4_ and 100 µM DPDP (3) and the spectrum of 100 µM K_2_PtCl_4_ + 100 µM DPDP (4) indicates that Pt^2+^ was bound to DPDP, since the spectrum after incubation differs from the sum of the spectra of the two particular substances. The main difference is that the UV-absorption maximum at 255 nm increases substantially (2.5 times) after incubation. This indicates that the UV-absorption maximum at 255 nm is most likely related to the metal ion bound to DPDP. Qualitatively the spectra of 100 µM K_2_PtCl_4_ + 100 µM DPDP (4) and 100 µM K_2_PtCl_4_ + 100 µM MnDPDP (5), are rather similar, while they both differ significantly from the 100 µM MnDPDP spectrum (6). Firstly, the main UV-absorption maximum in PtDPDP (located at 207 nm) has blue-shifted compared to the 220 nm in MnDPDP. Secondly, the maximum at 319 nm in MnDPDP has been replaced by two peaks located at 296 and 330 nm. These results indicate that in the competition experiments between K_2_PtCl_4_ + MnDPDP almost all of the Mn^2+^ was replaced by Pt, which confirms the EPR results. The additional weak feature at 380 nm in 100 µM K_2_PtCl_4_ + 100 µM MnDPDP (5) is related to MnO_2_^23^. Other manganese oxides were probably formed as well and added most likely a weak and broad background signal which makes all the peaks appear broadened^24^. This also indicates that Mn^2+^ in DPDP was replaced by Pt^2+^.

## Discussion

Oxaliplatin, a third-generation platinum agent, is considered one of the two most important new drugs used in the treatment of CRC^25^. Despite the clinical impact and enthusiasm for this agent, the applicability of oxaliplatin is limited, in a large part, by CIPN and myelosuppression. Importantly, chronic neurotoxicity, and not tumor progression, is the most frequent reason that forces patients to forego further therapy with this agent. Because the approved use of oxaliplatin has, for the first time in over 50 years, improved overall survival in patients with CRC, innovative research with respect to oxaliplatin-induced CIPN is highly appreciated^25^.

Clinical data indicate an apparent lack of correlation between Pt^2+^-induced neurotoxicity and tumor response^26^, suggesting that neurotoxicity and other adverse events may be prevented without altering tumoricidal efficacy. Furthermore, after administration into patients, oxaliplatin (diaminocyclohexane platinum oxalate) is rapidly metabolized (plasma half-life less than 30 minutes) into numerous metabolites and less than 3% of it is converted into the tumoricidal active metabolite diaminocyclohexane platinum dichloride [Pt(dach)Cl_2_]^27^ that after sequential replacement of the chlorides with water is a key step in forming a complex with the DNA^26^. It is hence reasonable to anticipate that it is the non-active Pt^2+^- metabolites that cause oxaliplatin-associated CIPN^27^, and that it is this large fraction of oxaliplatin-derived Pt^2+^ that is the presumptive target for DPDP/CaDPDP. An anticipation that together with preclinical and clinical data showing no negative influence of either MnDPDP or Ca_4_Mn(DPDP)_5_ on the tumoricidal activity of oxaliplatin (see Introduction), apparently suggest that DPDP/CaDPDP does not compete with the Pt^2+^-ligand diaminocyclohexane oxalate for binding Pt^2+^. The rapid metabolism of oxaliplatin suggests that even co-administration of DPDP/CaDPDP and oxaliplatin will cause little or no chelation of oxaliplatin-bound Pt^2+^.

The exact mechanism behind chronic oxaliplatin-associated CIPN is, like that of cisplatin, poorly understood but is apparently caused by increase in oxidative and nitrosative stress in neurons of the dorsal root ganglion (DRG)^1^. Mechanistic studies suggest a role of transport mechanisms in Pt^2+^ -induced neurotoxicity^28^. Sprowl *et al*.^28^ reported that accumulation of oxaliplatin-associated Pt^2+^ in, and damage to, neuronal cells is linked to the organic cation transporter (OCT2), a protein expressed in the neurons of the DRG. Overexpression of OCT2 results in a significant (up to 35-fold) increase in neuronal uptake of oxaliplatin-associated Pt^2+^, while OCT2 gene knockout protected against the development of peripheral neurotoxicity.

**Figure 5:**
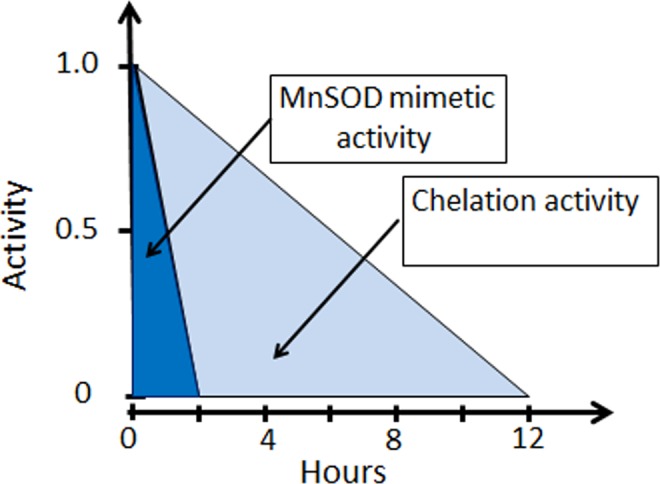
The two therapeutic mechanisms of MnDPDP and Ca_4_Mn(DPDP)_5_, i.e., MnSOD mimetic activity and metal chelation activity of DPDP/CaDPDP, and the meaning of them during acute increase in oxidative/nitrosative stress and that caused by a prolonged increase in oxidative/nitrosative stress, such as from retention of Pt^2+^ in the body. During the acute increase in the cellular stress, MnSOD mimetic activity is the most important mechanism to balance the cellular stress burden, although metal chelation also plays an important role. Pharmacokinetic behavior of MnDPDP in healthy volunteers suggests that the MnSOD mimetic activity of MnDPDP last no longer than 2 hours post-injection, whereas the chelation activity lasts about 12 hours^15^. During prolonged increase in cellular stress, causing irreversible inactivation of endogenous mitochondrial MnSOD (the most important endogenous cellular defensive against increased oxidative/nitrosative stress^2^), metal chelation is probably the main mechanism to balance the burden, such as that caused by retention of the transitional metal Pt^2+^, where chelation of Pt^2+^ may be of a particularly importance.

The recent finding by Coriat *et al*.^4^ that MnDPDP has a curative effect on chronic oxaliplatin-associated CIPN through its MnSOD-mimetic activity is from a pharmacokinetic perspective questionable. An until recently undisclosed possibility^3^ is that Pt^2+^ outcompetes Mn^2+^ for binding to DPDP (or its metabolites) and transforms toxic Pt^2+^ into a non-toxic complex, which can be readily excreted through the kidneys^3^, by a process known as chelation therapy. Retention of Pt^2+^ within DRG nerve cells presumably causes a long-lasting increase in oxidative and nitrosative stress, where a few hours increase in MnSOD mimetic activity is not expected to have any major curative effect. The various therapeutic activities of MnPLED-derivatives and the meaning of them during acute and prolonged increase in oxidative/nitrosative stress, respectively, are schematically illustrated in Fig. 5.

To be efficacious as therapy against Pt^2+^ intoxication, as well as other metal intoxication, a chelator has to satisfy criteria that allows it to^29,30^: (i) transport across physiological barriers into compartments where a toxic metal ion is concentrated, (ii) form a stable complex with the metal after removing it from the compartments and (iii) form a chelation complex whose properties render it non-toxic and facilitate its excretion.

Chelation therapy has been tested regarding cisplatin-associated renal toxicity in a couple of animal studies^31^. These studies have demonstrated that various sulfur-containing chelators may protect against cisplatin-associated renal toxicity without affecting antitumor activity significantly. The sulfur compound (diethyldithiocarbamate; DDTC) was tested in a randomized placebo-controlled trial in 221 patients receiving cisplatin (100 mg/m^2^) combined with cyclophosphamide or etoposide^32^. DDTC did not reduce the incidence of peripheral neurotoxicity. On the other hand, renal toxicity and treatment withdrawal due to toxicity was significantly higher in the DDTC group than in the control group, presumably illustrating the problem of finding or creating an ideal chelator. To the best of our knowledge, oxaliplatin with a completely different pharmacokinetic profile in comparison to cisplatin^33^ has never been subject for chelation therapy in order to treat oxaliplatin-associated adverse events.

The present study suggests that Pt^2+^ has a higher affinity than Zn^2+^ for DPDP i.e., a ^10^logK_ML_ higher than 19; the EPR-guided competition experiments do not tell how much higher but it may be considerably higher than that of Zn^2+^. Pearson^34^ categorized acids (metal cations) and bases (chelators) as either hard or soft. Hard acids tend to bind to hard bases and soft acids tend to bind to soft bases. Preferred endogenous binding sites for soft and borderline metal ions such as Pt^2+^, Cu^2+^ and Zn^2+^are thiolates, amides^29^ and phenolates^17^.

The results of the present study provide evidence that Pt^2+^ has an affinity for DPDP at least 10,000 times higher than that of Mn^2+^. Borderline Cu^2+^ has a high affinity for DPDP with a ^10^log K_ML_ of 22.08^17^, which is about 1000 times higher than that of Zn^2+^.The ^10^log K_ML_ of MnDPDP is15.10^17^. Whereas Mn^2+^ binds with six coordinates to two phenolates, two amides and two carboxylates of DPDP or its metabolite PLED (Fig. 1), Cu^2+^ binds with 4 coordinates to the amides and phenolates, in a square pyramidal geometry^16,17^. Because of the smaller ionic radius of Cu^2+^ this metal ion forms a much more stable complex, with shorter bond distance^18^, than that found for the Mn^2+^ ion^16^. Similarities between Cu^2+^ and Pt^2+^ (see Introduction) suggest that Pt^2+^ also binds with high affinity to DPDP. The present results show that Pt^2+^ has an affinity for DPDP higher than that for Zn^2+^, suggesting that this chelator may be ideal for chelation therapy during chronic CIPN caused by oxaliplatin and other Pt^2+^-containing chemotherapy drugs. Multiple lines of evidence indicate that Pt^2+^-containing cancer drugs enter cells and are exported from cells via transporters that evolved to manage copper homeostasis^35^, probably related to the above mentioned similarities in coordination geometry between Pt^2+^ and Cu^2+^.

Metal complexes of DPDP (or its metabolites) are excreted through the kidneys by a glomerular filtration rate-governed process^15^. The pharmacokinetics of Pt^2+^- metabolites of oxaliplatin in plasma is typically triphasic in man, characterized by a short initial distribution phase and a long terminal elimination phase with a half-life of about 11 days. Anticipating that 5 half-lives are needed to reach the zero plasma level of Pt^2+^, this means that it will take around 55 days to get all Pt^2+^ out of the body. Curative treatment in CRC patients with oxaliplatin (in combination with 5-fluorouracil) following the FOLFOX regime typically include 12 cycles every 14 days. The pharmacokinetics clearly suggest an increasing burden of Pt^2+^ over the whole treatment period. The long terminal half-life probably represents a slow release of low molecular weight platinum-amino acid conjugates after degradation of cellular macromolecules^33^. It seems plausible that DPDP (or its metabolites) may increase Pt^2+^-elimination during the long elimination phase by having a high enough affinity for Pt^2+^, which in turn may increase renal excretion of Pt^2+^ substantially. Such a process may explain the apparent curative effect of MnDPDP on oxaliplatin-associated CIPN. This effect is expected to be amplified in the case of Ca_4_Mn(DPDP)_5_ (calmangafodipir) due to its 4 time higher content of DPDP at equimolar concentrations of Mn^2+^ (see below and Fig. 1), where Ca^2+^ has about 10,000 time lower affinity for DPDP^21^ than Mn^2+^.

Both essential and nonessential metals may exert toxic effects if the dose of exposure exceeds certain levels^36^. Manganese is essential in a number of enzymes, of which MnSOD is of particular relevance, since it protects mitochondria from toxic oxidants. Overexposure to manganese may give rise to “manganism” with Parkinson-like symptoms. Mn^2+^ bound to DPDP is likely non-neurotoxic, but Mn^2+^ released from DPDP may pass the blood-brain-barrier and accumulate in the brain, causing severe neurotoxicity^1,2^. About 80% of the Mn^2+^ content of MnDPDP is released upon intravenous injection of a clinically relevant dose^15^. It is possible to stabilize MnDPDP by replacing 4/5 of its Mn^2+^ content with Ca^2+^, resulting in a compound known as calmangafodipir [Ca_4_Mn(DPDP)_5_]^2,14^. At equivalent intravenous Mn^2+^ doses, Ca_4_Mn(DPDP)_5_ causes considerably less Mn^2+^ release and retention in the rat brain and is significantly more efficacious than MnDPDP to protect mice against oxaliplatin-induced leukocytopenia. Although brain retention of manganese is reduced by about 40%, it still occurs and restricts the use of Ca_4_Mn(DPDP)_5_. Standard chemotherapy with 5-fluoruracil plus oxaliplatin (FOLFOX) in stage III CRC patients involves 12 consecutive cycles, although the number of cycles may be reduced down to 6 in low-risk stage III CRC patients^37^. From a risk-benefit perspective it seems reasonable to include co-administration of 5 µmol/kg Ca_4_Mn(DPDP)_5_ in each of these cycles, in order to lower the incidence of acute CIPN and other dose limiting toxicity, such as severe leukocytopenia and mucositis. For more frequent use of Ca_4_Mn(DPDP)_5_, accumulated Mn^2+^ neurotoxicity will most probably represent an insurmountable obstacle. With regards to Pt^2+^-associated chronic CIPN, the possibility of using “naked” DPDP or CaDPDP may offer a considerable improvement to treat this serious condition. Furthermore, use of non-Mn^2+^ containing DPDP (or its dephosphorylated counterpart PLED), in addition, offers an attractive possibility to treat oxaliplatin-associated chronic CIPN temporarily separated from oxaliplatin administration. Such an approach will enable repeated administration of the chelator over days or weeks in order to transport as much Pt^2+^ as possible away from the body.

MnDPDP has been used as an MRI contrast agent in more than 100,000 patients and repeated dosing of Ca_4_Mn(DPDP)_5_ has been tested in about 100 patients as an adjunct to chemotherapy, both compounds have been well tolerated^5,13^. Taking into consideration that it is the toxicity of manganese that limits the use of these compounds, it should be possible to proceed directly to a study with a limited number of patients with chronic oxaliplatin-associated CIPN and test manganese-free DPDP or CaDPDP in a manner similar to that described by Coriat *et al*., i.e., in patients with severe CIPN (grade ≥ 2 on a 3 graded scale)^4^. The extremely high sensitivity and precision of inductively coupled plasma mass spectrometry (ICP-MS)^33^ will allow analyses of Pt^2+^ in the urine after administration of DPDP or placebo in patients with chronic oxaliplatin-associated CIPN. Preclinical studies demonstrate that MnPLED-derivatives have a tumoricidal activity of its own^1,2,12,38,39^. Furthermore, the tumoricidal activity of MnPLED-derivatives seems to be a property of the chelator alone and not a property of the intact Mn(II)complex^11,14^, which in fact may contribute to increase survival among the CRC patients.

## Conclusion

In conclusion, the present study shows that Pt^2+^ has an affinity for DPDP statistically significantly higher than that for Zn^2+^. ZnPLED is the main metabolite found in the urine after administration of DPDP, CaDPDP or MnDPDP^15^ suggesting that DPDP or its metabolites DPMP and PLED may form stable enough metal complexes for fulfilling the above-mentioned criteria. This together with rather rapid excretion kinetics of metal(II)DPDP/PLED^15^ and low toxicity, speaks in favour of using DPDP or CaDPDP as a chelation therapeutic drug in conditions of chronic Pt^2+^ intoxication (Fig. 5), such as that seen after chemotherapy with oxaliplatin and cisplatin.

## Methods

### Chemicals and reagents

Mangafodipir trisodium (MnDPDP) and fodipir (DPDP) (Fig. 1) were gifts from Nycomed Imaging/GE Healthcare (Oslo, Norway). Potassium tetrachloroplatinate(II) (K_2_PtCl_4_), MnCl_2_ and ZnCl_2_ were purchased from Merck Sweden.

### Sample preparations

Prior to the experiments, the chemicals were dissolved in water to suitable strengths. The pH of 1.0 wt% MnDPDP trisodium in water is in the range of 6.36–6.77^17^. MnDPDP trisodium has a considerable buffering capacity as the pH for 1.0 wt% MnDPDP trisodium solution in 1.0 mM HCl and 1.0 mM NaOH were found to be 6.31–6.32 and 6.37–6.40, respectively. Making use of the high buffering capacity of MnDPDP trisodium, there was no need for using any other buffer, which is of course a great advantage when conducting EPR and optical absorption spectroscopy. Rechecking the samples after conducting the experiments showed pH just below 7.

### Physical background

Mn^2+^ has five unpaired electrons in a (3d^5^) configuration, with its electronic ground state in the octahedral ligand field being in the (^6^S) high-spin configuration with an electron spin *S = *5/2. It can be described by a spin-Hamiltonian including an electron Zeeman term, a zero field splitting (ZFS) term, a central hyperfine interaction term, the quadrupole interaction term and a nuclear Zeeman term, in the following form:

1 $$ {\mathcal H} ={\mu }_{B}{\boldsymbol{BgS}}+{\boldsymbol{SDS}}+{\boldsymbol{SAI}}+{\boldsymbol{IQI}}-{g}_{N}{\mu }_{N}{\boldsymbol{BI}}$$

Here, B is the external magnetic field, **S** denotes an effective electron spin, **I** is the nuclear spin and µ_B_ and µ_N_ is are the Bohr- and nuclear magneton, respectively. **g** and g_N_ are the electron and nuclear g-tensor, respectively. **A** is the hyperfine interaction tensor. The ZFS for S > 1/2 is described by the tensor **D**. The quadrupole interaction for nuclei with I > 1/2 is given by the tensor **Q**.

In liquid solutions, i.e., hexaqua-Mn^2+^ can be described by just isotropic interactions, since it is subject to fast rotational and tumbling motion, resulting in all anisotropic interactions in the spin-Hamiltonian being averaged out. Therefore, the EPR signal from Mn^2+^ ions in liquid solutions consist of six lines centered at g = 2.0, which are split by isotropic hyperfine interaction with the ^55^Mn nucleus (I = 5/2 with 100% natural abundance). Due to the S = 5/2 electron spin multiplet of Mn^2+^, each of these six hyperfine lines consists of five overlapping electron spin transitions, which occur between adjacent M_S_ =  ± 5/2, ± 3/2, ± ½ sublevels (M_S_ is the electron spin projection). Each of these transitions is weighted by its respective transition probability, $S(S+1)-{M}_{S}({M}_{S}+1)$, and, thus, contributes non-equally to the EPR signal intensity^40^. Additionally, incomplete motional averaging of the anisotropic interactions (like fine structure splitting and anisotropic hyperfine interactions) can cause M_S_-dependent broadening^41^. Therefore, the transitions involving higher M_S_ states are usually broader than transitions between the inner states M_S_ =  ± 1/2. Such a non-equivalent broadening yields additional weighting of the transitions so that broader transitions, i.e. those with higher M_S_, have smaller peak-to-peak intensity, as it is observed in this case.

However, ligand-bound Mn^2+^ (like in MnDPDP) is, unlike hexaqua-Mn^2+^, subject to slow tumbling processes, which causes a broadening of the EPR signal, which makes it undetectable in the micromolar concentration range in X-band EPR^21^. These differences can be used to monitor the Pt^2+^/Mn^2+^ and Zn^2+^/Mn^2+^ exchange by detecting free Mn^2+^ ions.

### Competition experiments between MnDPDP and ZnCl_2_ or K_2_PtCl_4_ evaluated by EPR spectroscopy

Pilot experiments indicated that complete Pt^2+^/Mn^2+^ exchange was obtained after 5 days of incubation at room temperature. The long incubation time needed is presumably due to slow Pt^2+^ exchange kinetics^19,20^ and steric hindrance in the hexa-dendate MnDPDP complex. The steric hindrance of naked DPDP or CaDPDP (where Ca^2+^ is loosely bound to DPDP) is anticipated to be considerably lower. When incubated at 60 °C complete exchange was obtained within 4 h. Anticipating that the reaction rate will double for every 10 °C, 4 h at 60 °C corresponds to about 1 day at 37 °C. Competition experiments were conducted between a fixed concentration of 100 µM MnDPDP and 0–1000 µM ZnCl_2_ or K_2_PtCl_4_. All samples, except those of 300 and 1000 µM were incubated 6 h at 60 °C; the two highest concentrations were incubated 18 hours in order to make sure that the curves saturated at 100 µM ZnCl_2_/K_2_PtCl_4_.

X-band EPR spectra (9.9 GHz) of Mn^2+^ were recorded on a Bruker Elexsys E500 spectrometer at room temperature making use of Pasteur pipets and the capillary action of their tips, allowing a sample size of 100 µL. Standard measurement parameters were 100 kHz modulation frequency, 10 G modulation amplitude, 12 mW microwave power, and 65 G/s sweep velocity. Signal intensity was determined by double integration after baseline correction, and each EPR session was normalized against the standard curve (Fig. 2).

### Optical absorption spectroscopy

Optical absorption spectra in a spectral range from 200–1000 nm were recorded with a Shimadzu UV-2450 spectrometer.

### Statistical analyses of competition curves

Competition curves for ZnCl_2_ and K_2_PtCl_4_ were analyzed by fitting the experimental data into the sigmoidal response logistic equation (GraphPad Prism, version 6.0, La Jolla, CA):

2 $$[M{n}^{2+}]={[M{n}^{2+}]}_{\max }\cdot \frac{{[P{t}^{2+}]}^{q}}{{[P{t}^{2+}]}^{q}+{[M{n}^{2+}]}_{\max }^{q}}$$

by means of non-linear, least squares regression analysis, where Mn^2+^, Mn^2+^_max,_ Pt^2+^ denote the observed concentration of free Mn^2+^, maximum concentration of free Mn^2+^, and the concentration of Pt^2+^, respectively. Mn^2+^_50_ defines the concentration of Pt^2+^ causing half-maximal increase in free Mn^2+^, and the constant q determines the slope of the curve. The same function was also applied when analyzing the competition curve between MnDPDP and Zn^2+^. From this analysis, the E_max_ (maximal response) and the pD_2_ [−^10^log of the concentrations (M) of a drug causing half maximal responses; EC_50_] were calculated. E_max_ (µM) and pD_2_ values are presented together with 95% confidence intervals.
